# Detailed mapping of mesothelioma cases in Denmark to identify areas with elevated risk: a nationwide population-based study

**DOI:** 10.5271/sjweh.4229

**Published:** 2025-09-01

**Authors:** Heidi Søgaard Christensen, Rikke Hedegaard Jensen, Lars Hernández Nielsen, Lise Dueholm Bertelsen, Christian Teglgaard, Jakob Hjort Bønløkke, Marianne Tang Severinsen, Martin Bøgsted

**Affiliations:** 1 Center for Molecular Prediction in Inflammatory Bowel Disease, Department of Clinical Medicine, Aalborg University, Aalborg, Denmark.; 2 Center for Clinical Data Science, Department of Clinical Medicine, Aalborg University, Aalborg, Denmark.; 3 Clinical Cancer Research Center, Aalborg University Hospital, Aalborg, Denmark.; 4 Department of Clinical Medicine, Aalborg University, Aalborg, Denmark.; 5 Danish Center for Health Services Research, Department of Clinical Medicine, Aalborg University, Aalborg, Denmark.; 6 Department of Haematology, Aalborg University Hospital, Aalborg, Denmark.; 7 Department of Occupational and Environmental Medicine, Aalborg University Hospital, Aalborg, Denmark.

**Keywords:** disease hotspot, disease mapping, malignant mesothelioma, rare disease, spatial analysis

## Abstract

**Objectives:**

Previous studies mapping pleural mesothelioma in Denmark have found that the risk varies between Danish regions. However, evaluating disease risk for such relatively large geographical units ignores any heterogeneity within the unit and can thus diminish more local spikes in risk, missing smaller areas of excess risk. In this study, we examined the distribution of pleural mesothelioma in Denmark on an unprecedented detailed scale, mapping cases to each of the Danish parishes.

**Methods:**

We identified individuals diagnosed with pleural mesothelioma between 1990 and 2021 in the Danish Cancer Registry. Considering age- and sex-standardized incidence rate ratios (IRR), we used a conditional autoregressive random effects model to smooth IRR across parishes. Parishes with a smoothed parish-to-national IRR >1.25 or 2.0 with a posterior probability of >95% were flagged as parishes with an excess risk of pleural mesothelioma.

**Results:**

We identified 3105 incident cases of pleural mesothelioma in the study period. A total of 74 and 14 parishes were flagged with IRR significantly above 1.25 and 2.0, respectively. These parishes had posterior mean smoothed IRR of 1.82–4.13.

**Conclusions:**

We provided a detailed mapping of pleural mesothelioma cases in Denmark and found five distinct areas, each covering several parishes, with a significantly elevated risk. All these areas were in the proximity of previous asbestos-using industries.

Mesothelioma is a rare malignancy with a high mortality rate that is most commonly found in the pleura (1). At least 80% of pleural mesothelioma cases can be linked to prior exposure to asbestos (1). Studies mapping mesothelioma cases have identified an elevated risk in areas of previous asbestos-using industries (2–5). In Denmark, reported differences in incidence rates of mesothelioma across the five Danish regions are presumably also associated with the location of previous asbestos-using industries (6–8). However, Danish regions are relatively large geographical units, containing 10–30% of the Danish population, and thus more local spikes in risk were diluted. Panou et al (4) did provide a more granular mapping of mesothelioma in Denmark, but only for women in the North Denmark region and with a focus on distinguishing environmentally and occupationally caused cases.

In this paper, we present an unprecedented detailed mapping of all incident pleural mesothelioma cases in Denmark from 1990 to 2021 across the 2141 Danish parishes.

## Methods

### Danish mesothelioma data

Danish residents diagnosed with pleural mesothelioma between 1 January 1990 and 31 December 2021 were identified using the Danish Cancer Registry, which contains information on all cancer cases in the Danish population since 1943 (9). Diagnoses are coded according to the International Classification of Disease 10^th^ Revision (ICD-10) or converted to ICD-10 if given before ICD-10 were introduced, and morphology is coded according to the International Classification of Diseases for Oncology 3^rd^ Revision (ICD-O-3) (9). We defined pleural mesothelioma cases by either ICD-10 code C45.0 or ICD-10 code C38.4 with ICD-O-3 morphology codes indicating mesothelioma (M9050/3, M9051/3, M9052/3, or M9053/3). Individuals were assigned to a parish based on residential address at diagnosis using the parish division as of 2021; parish populations ranged from 31 to 46 586 individuals with a median population of 1039 individuals.

This study is a register study that does not require informed consent in Denmark. The study has, according to the General Data Protection Regulation (GDPR), been registered in the North Denmark region’s list of register studies (reg. no. F2023-095).

### Statistical analysis

We calculated the parish-to-national incidence rate ratio (IRR) using indirect standardization with respect to age and sex, considering seven age groups (≤40, 41–50, 51–60, 61–70, 71–80, 81–90, and >90 years).

As the number of cases per parish is low, the raw incidence rate estimates are unstable. Therefore, we considered a model framework that allowed us to improve estimates of the IRR by borrowing information from neighboring parishes and thus smoothing estimates across parishes. Specifically, using a fully Bayesian setup, we modelled the number of cases by a spatial Poisson distributed generalized linear mixed model with a conditional autoregressive (CAR) correlation structure specified by the Leroux model (10); details can be found in the supplementary material, www.sjweh.fi/article/4229, appendix A. For each parish, we calculated the posterior probability of the smoothed IRR being >1.25 and 2, corresponding to incidence rates elevated by >25% or 100%, respectively, compared to the national rate. Parishes with a posterior probability >95% were flagged as areas with elevated risk of pleural mesothelioma at the specified level. Residual autocorrelation was evaluated using Moran’s I statistic.

Analyses were performed in R (v4.2.2) (11) using the R-package *CARBayes* (v6.1.1) to fit the spatial model and *ape* to calculate Moran’s I statistic (v5.8). Code used to run the analysis is exemplified on github.com/CLINDA-AAU/disease_CAR_example.

## Results

A total of 3105 Danish residents were diagnosed with pleural mesothelioma between 1 January 1990 and 31 December 2021, corresponding to a national incidence rate of 1.81 per 100 000 person-years. Of these, 2618 (84%) were male and the overall median age at diagnosis was 70 years. The national incidence rate was highest among individuals aged 70–90 and substantially higher among males than females across all age groups (see table 1).

**Table 1:** The national incidence rate per 100 000 person-years of pleural mesothelioma for each age and sex group.

Age group (years)	Male incidence rate	Female incidence rate
≤40	0.02	0.02
41−50	0.67	0.17
51−60	3.30	0.63
61−70	9.48	1.53
71−80	16.84	2.16
81−90	14.13	2.22
>90	3.26	1.01

**Figure 1:**
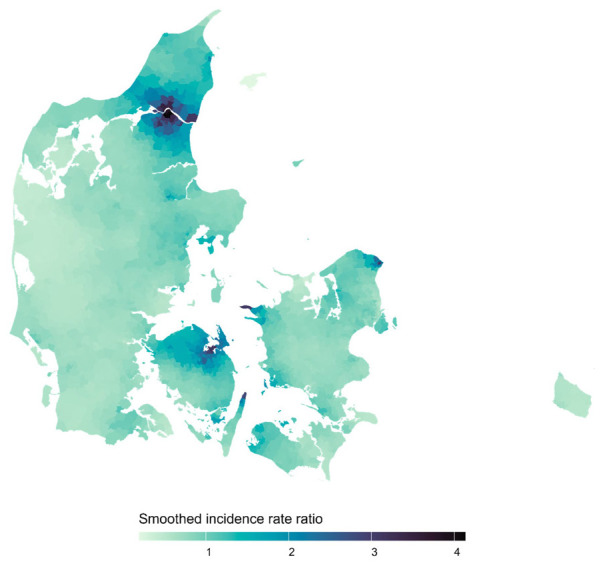
Posterior mean of the smoothed parish-to-national incidence rate ratios of pleural mesothelioma in Denmark for each parish.

The raw age- and sex-standardized parish-to-national IRR are shown in supplementary figure B1 in appendix B, and the posterior mean of the smoothed IRR obtained from the fitted CAR model in figure 1 below. No residual autocorrelation was detected (Moran’s I statistic= -0.014, P-value=0.33).

**Figure 2:**
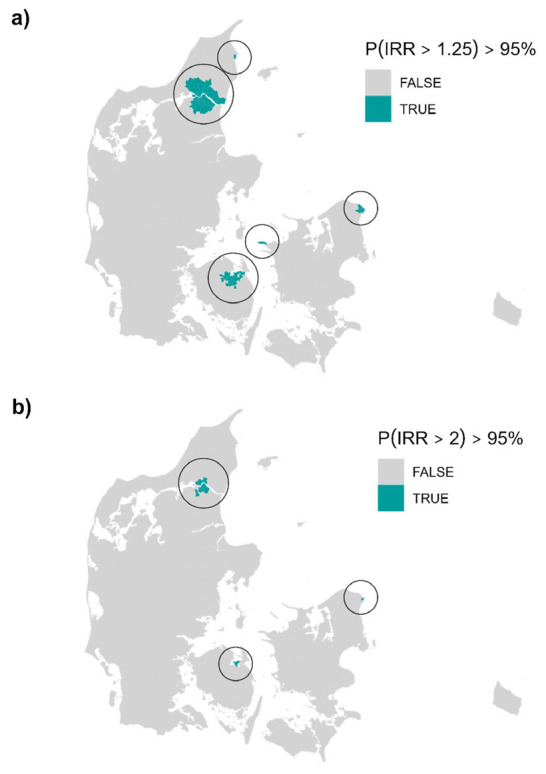
Parishes with smoothed incidence rate ratios (IRR) >25% (a) and 100% (b) higher than the national level with a posterior probability >95%.

We found 74 and 14 parishes with smoothed incidence rates >25% and 100%, respectively, above the national with a posterior probability of >95% (see figure 2 as well as supplementary figure B2 and table B1 in appendix B). The parishes flagged with an incidence rate elevated by >100% had posterior mean smoothed IRR of 2.85–4.13 and were located in three distinct areas of Denmark around the cities of Aalborg, Odense, and Helsingør. Considering an elevated risk of >25%, more parishes in these three areas were flagged as well as one parish near Kalundborg and one in Frederikshavn; the posterior mean smoothed IRR were 1.83–4.13.

At diagnosis, individuals in our cohort had on average lived at the same address for 27 years.

## Discussion

We found five distinct areas in Denmark that with high certainty had an elevated risk of pleural mesothelioma. Specifically, 74 parishes in five distinct areas were flagged with an incidence rate elevated >25%, while 14 parishes in three distinct areas were flagged with smoothed incidence rates more than twice the national level. The 14 parishes were located around and in the cities of Aalborg, Odense, and Helsingør, while the 74 parishes further involved Frederikshavn and Kalundborg. All these cities previously had shipyards, and Aalborg further had a large asbestos cement factory, Odense a large producer of asbestos-containing brakes, and Kalundborg an oil refinery. Workers in such industries are of increased risk of mesothelioma (12–14). It should be noted that asbestos use has, with few exceptions, been prohibited in Denmark since 1986, though the use of asbestos in brakes were not prohibited until 2004 (15).

In agreement with our results, previous studies report increased standardized IRR for male inhabitants in several Danish cities with previous shipyards and/or an asbestos cement factory with the highest ratios in Aalborg, Helsingør, and Odense (6, 16). With our choices of thresholds, no parishes were flagged in the capital area of Copenhagen, although existing studies, considering earlier time periods, have reported an increased risk (6, 7, 16). As noted by Skammeritz et al (7), the male incidence rate of mesothelioma in the capital region of Denmark seems to have stagnated since the 1980s, while incidence rates in the North Denmark region and the South Denmark region, that contain the cities Aalborg and Odense, respectively, have continued to increase up until 2009 where their study period ended (7). Since we examined the period from 1990 to 2021, our study confirmed this decreased incidence rate of mesothelioma in the capital region.

Asbestos exposure among individuals with mesothelioma has been thoroughly examined in the area of Aalborg (4, 17–19). Among 122 patients seen at an occupational clinic in Aalborg, 87.7% had a known occupational exposure (18). For female patients in the North Denmark region, 75% had an identified asbestos exposure with non-occupational exposure accounting for the majority of cases (4). Additionally, increased risks of mesothelioma have been reported among individuals who as children attended schools and lived in the neighborhood of the asbestos cement factory in Aalborg (19).

In this study, mapping was based on the residential location at diagnosis. However, due to the long latency period from first asbestos exposure to development of pleural mesothelioma with a median of 38 years, the distribution at time of diagnosis does not necessarily reflect areas of asbestos exposure (20). This is evident in an Australian study where high counts of cases were seen in popular retirement areas in Perth while only few cases were reported in previous mining and milling areas that have been depopulated since the industry closed down (5). At diagnosis, individuals in our cohort had on average lived at the same address for 27 years. Note, other choices of baseline incidence rate than the national or other probability thresholds may lead to different results. In this study, we displayed the geographical heterogeneity of pleural mesothelioma risk, but underlying reasons for heterogeneity, including differentiation of occupational and environmental asbestos exposure, were not investigated.

A strength of our study is the validated data on diagnosis and parish of residence which have been retrieved from high-quality population-based registries. Furthermore, since the Danish healthcare system provides free access to health services, the risk of socioeconomic factors affecting the findings is considered low. Further, no existing study reports the risk of pleural mesothelioma at such granular level for all of Denmark and thus our study enables a more direct linkage of high-risk areas to the location of previous asbestos-using industries. In addition, our study showcases an approach to mapping rare diseases considering small geographical units and a well-known exposure-outcome pair in form of asbestos and pleural mesothelioma, and the statistical approach can be replicated for other diseases to generate hypotheses on geographically associated risk factors.

In conclusion, from a detailed mapping of pleural mesothelioma cases in Denmark, we found five distinct areas with elevated risk; all these areas could be linked to previous asbestos-using industries.

## Supplementary material

Supplementary material
