# Supplementary material for: Detailed mapping of mesothelioma cases in Denmark to identify areas with elevated risk: a nationwide population-based study
Source: Scand J Work Environ Health. 2025 Aug 29;51(5):449–53. doi: 10.5271/sjweh.4229 (PMC12415714; doi:10.5271/sjweh.4229)
Supplement: Supplementary material [file SJWEH-51-449-S001.pdf]

Detailed mapping of mesothelioma cases in Denmark to identify areas with elevated risk: a nationwide population-based study[1]

by Heidi Søgaard Christensen, PhD,[2] Rikke Hedegaard Jensen, MSc, Lars Hernández Nielsen, PhD, Lise Dueholm Bertelsen, MD, Christian Teglgaard, MD, Jakob Hjort Bønløkke, PhD, Marianne Tang Severinsen, PhD, Martin Bøgsted, PhD

1. Supplementary material
2. Correspondence to: Heidi Søgaard Christensen, Center for Molecular Prediction in Inflammatory Bowel Disease, Department of Clinical Medicine, Aalborg University, Aalborg, Denmark. [E-mail: hschr@dcm.aau.dk]

Appendix A: Workflow for identifying parishes of elevated risk

Standardised expected number of cases

To define which parishes have a significantly elevated risk of pleural mesothelioma, we consider the parish-to-national incidence rate ratio (IRR); that is, the ratio of the observed number of cases in a parish and the number of cases expected under the national incidence rate. As the incidence of pleural mesothelioma depends highly on age and sex, we consider the indirectly age- and sex-standardised expected number of cases. That is, for the *i*th parish we calculate the standardised expected number of cases by

$$e_i = \sum_j t_{ij} r_j,$$

where r_j is the national incidence rate for age and sex group *j* and t_{ij} is a measure of person-time in parish *i* and age and sex group *j*. We estimated person-years by $t_{ij} = \sum_k n_{ijk}$, where n_{ijk} is the population in parish *i* for age and sex group *j* in year *k* for *k* = 1990,..., 2021.

Note, due to a small number (or a zero-count) of age- and sex-specific observed cases of pleural mesothelioma in some parishes, we used indirect standardisation instead of direct standardisation which may be preferred if the counts are high enough (or at least non-zero) (1).

Spatial model

We considered a fully Bayesian model framework that allowed us to improve estimates of the IRRs by borrowing information from neighbouring parishes and thus smoothing estimates across parishes. Specifically, we considered a generalised linear mixed model with Poisson distributed response and a conditional autoregressive (CAR) prior proposed by Leroux et al. for modelling spatial autocorrelation (2). In detail, the model was given by

$$
\begin{aligned}
y_i|\lambda_i &\sim \text{Poisson}(\lambda_i),\\
\log(\lambda_i) &= \alpha + \log(e_i) + u_i,\\
u_i|u_{-i} &\sim \mathrm{N}\left(\frac{\rho\sum_j w_{ij}u_j}{\rho\sum_j w_{ij} + 1 - \rho}, \frac{\tau^2}{\rho\sum_j w_{ij} + 1 - \rho}\right),\\
\alpha &\sim N(\mu_\alpha, \sigma_\alpha),\\
\tau^2 &\sim \text{Inverse} - \text{Gamma}(a, b),\\
\rho &\sim \text{Uniform}(0, 1),
\end{aligned}
$$

where $y = y_1, \dots, y_m$ denotes the number of cases across the $m = 2141$ parishes, u_i a spatially autocorrelated random effect for parish *i*, u_{-i} all random effects except the *i*th, and $W = [w_{ij}]$ a neighbourhood matrix. We specified *W* as a binary matrix with $w_{ij} = 1$ if parish *i* and *j* shared a border or was connected by bridge, and, for parishes constituting a whole island, connections by ferry also qualified as a neighbour relation. Otherwise, $w_{ij} = 0$. The neighbour relations are illustrated in Figure A1. The model was fitted using the R-package CARBayes and its default settings for priors with $\mu_\alpha = 0$, $\sigma_\alpha = 100{,}000$, $a = 1$, and $b = 0.01$ (3).

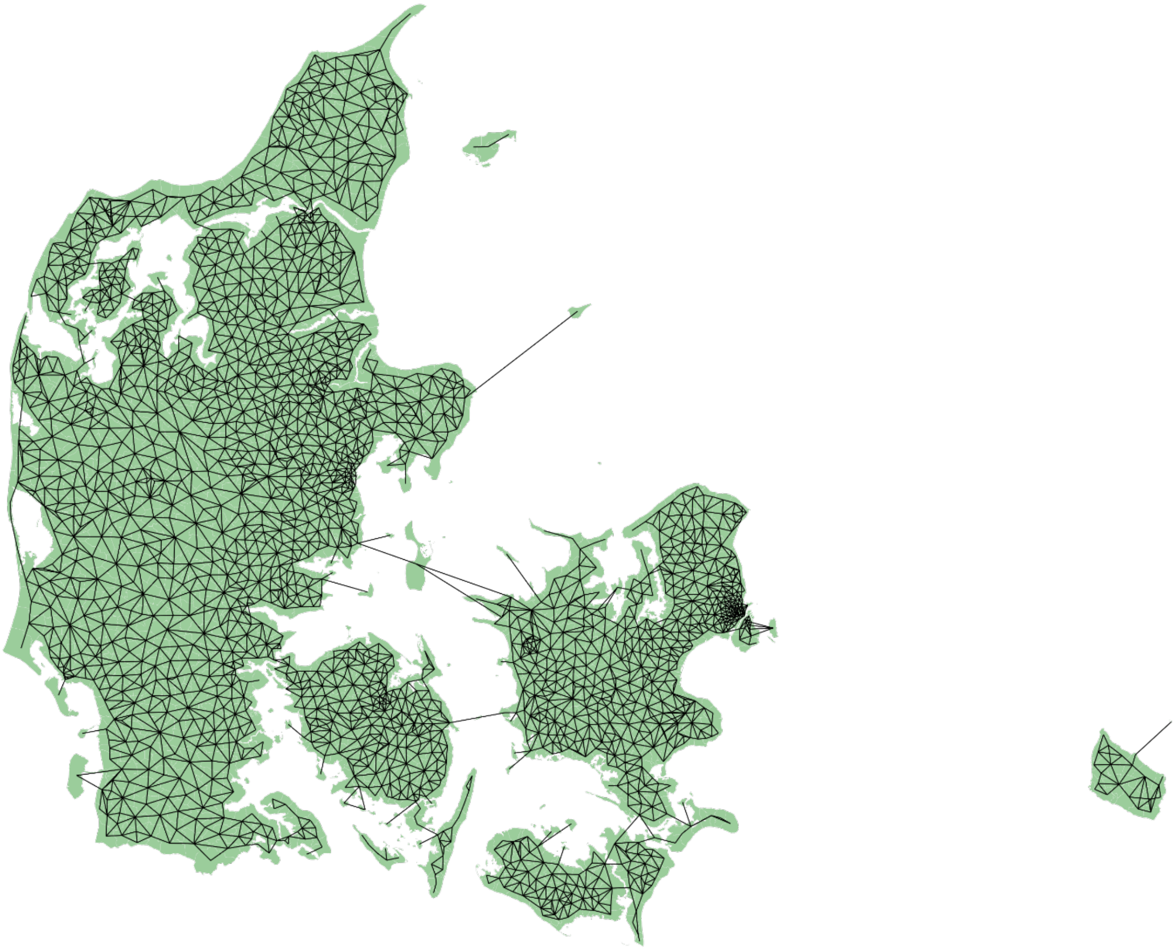

Figure A1: Illustration of the neighbour relations. Parishes connected by a line are considered neighbours

Flagging parishes with elevated risk

For a user-specified threshold $\theta > 0$, we consider incidence rates more than $100 * \theta\%$ above the national level to be elevated, and for the *i*th parish, we define its potential as an area of excess risk at level θ by

$$p_i(\theta) = P(\lambda_i/e_i \; > 1 + \theta \mid y)$$

the one-sided Bayesian credible interval of the smoothed IRR, λ_i/e_i. We flag the *i*th parish as having elevated risk at level θ whenever the posterior probability $p_i(\theta) > \omega$, where $0 < \omega \leq 1$ is a user-specified threshold.

In this study, we consider $\theta \in \{0.25, 1\}$ and $\omega = 0.95$.

Inference was based on Markov Chain Monte Carlo (MCMC) simulations from three chains, using a burnin of 100,000 samples, thinning of 20, and a total number of samples used for inference of 6,000 samples. For each parish *i*, we evaluated the posterior mean of the smoothed IRR λ_i/e_i and 95% credible intervals with limits given by the 2.5th and 97.5th percentiles. Further, we calculated the posterior probability $p_i(\theta)$, i.e., the proportion of MCMC iterations with $\lambda_i/e_i > 1 + \theta$.

Generalisability and code accessibility

The workflow described above to identify parishes with elevated mesothelioma risk was chosen to handle the low count of cases per parish and the smaller population in some parishes, leading to unstable incidence rate estimates. The method can be generalised to other diseases and geographical units. Note that administrative units like parishes usually change over time, and these changes need to be carefully tracked over the study period depending on the data available. An alternative would be to use a stationary geographical division, like the Danish national grid(4), if cases can be linked to each unit in the division and the population size per unit is known stratified on the selected age and sex groups.

Due to the GDPR, we are not permitted to publish the pleural mesothelioma data. However, to provide an example of the workflow our analysis is based on, R-code for analysing the distribution of males and females in Denmark has been made publicly available on GitHub: github.com/CLINDA-AAU/disease_CAR_example.

Appendix B: Result

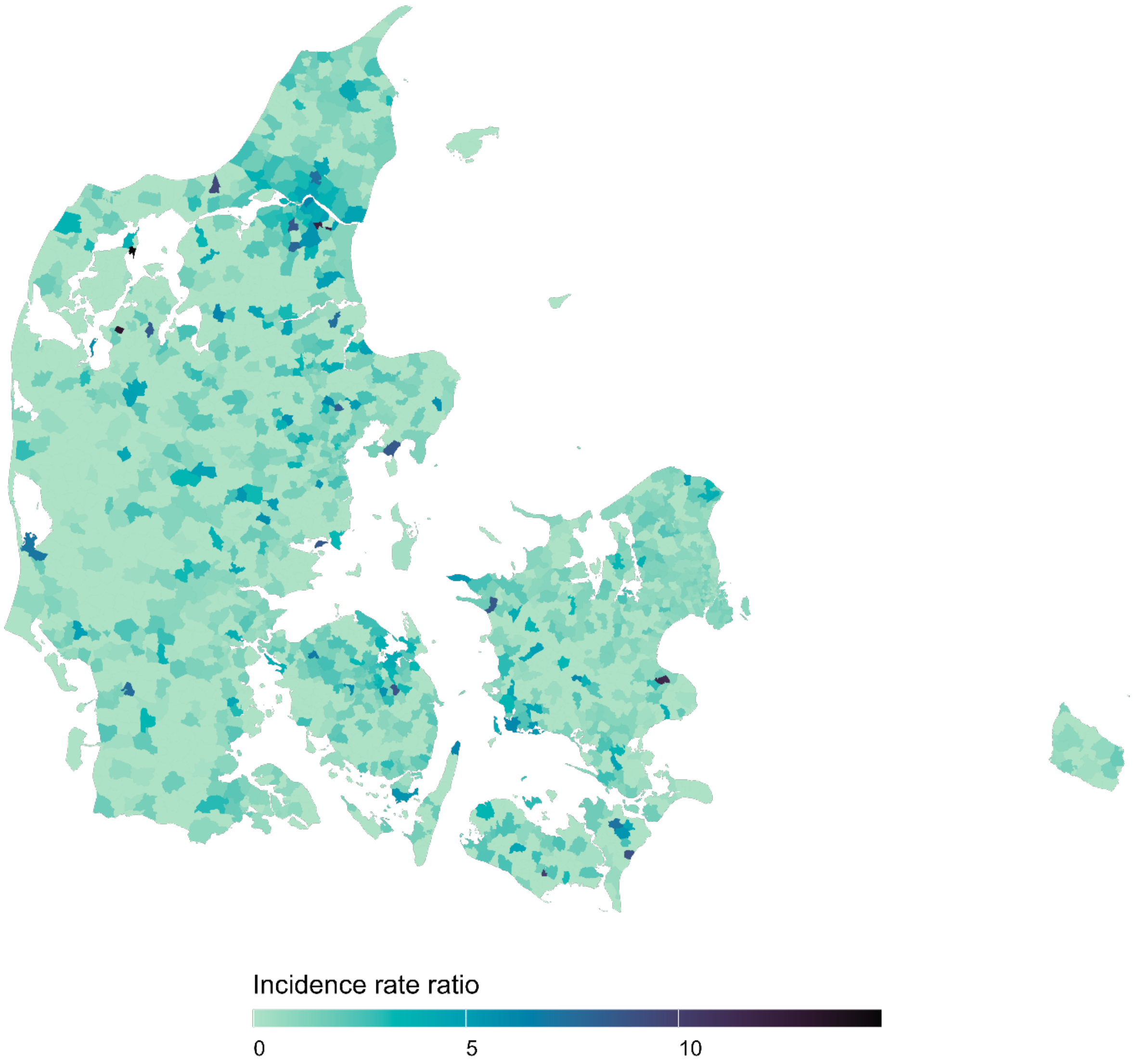

Figure B1: For each parish, the raw age- and sex-standardised incidence rate ratios (parish-to-national) of pleural mesothelioma

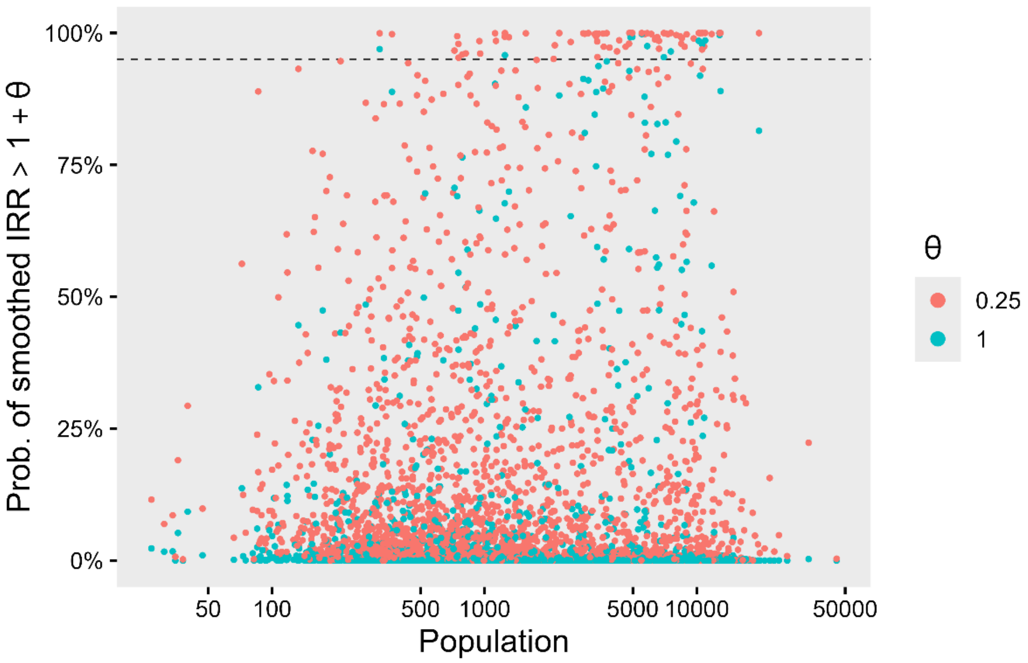

Figure B2: For each parish, the posterior probability that the smoothed incidence rate is more than 25% (red) or 100% (blue) higher than the national rate against the population size in 2021; the dashed line marks 95%

**Municipality**	**Parish**	**Smoothed IRR (95% CI)**
Aalborg	Ajstrup	1.95 (1.26, 2.88)
Aalborg	Godthåb	2.02 (1.16, 3.31)
Aalborg	Hasseris	2.08 (1.46, 2.84)
Aalborg	Ellidshøj	2.11 (1.27, 3.29)
Aalborg	Øster Hassing-Gåser	2.28 (1.31, 3.63)
Aalborg	Gudumholm	2.29 (1.33, 3.67)
Aalborg	Ansgars	2.29 (1.42, 3.44)
Aalborg	Ferslev	2.32 (1.32, 3.83)
Aalborg	Horsens	2.34 (1.40, 3.65)

Aalborg	Budolfi	2.38 (1.52, 3.49)
Aalborg	Frejlev	2.41 (1.45, 3.79)
Aalborg	Vadum	2.45 (1.57, 3.61)
Aalborg	Nørresundby	2.49 (1.52, 3.75)
Aalborg	Gunderup	2.53 (1.66, 3.71)
Aalborg	Sejlflod	2.56 (1.20, 4.77)
Aalborg	Nøvling	2.63 (1.69, 3.87)
Aalborg	Margrethe	2.63 (1.69, 3.85)
Aalborg	Svenstrup	2.67 (1.67, 4.01)
Aalborg	Vester Hassing	2.69 (1.58, 4.26)
Aalborg	Sulsted	2.77 (1.77, 4.14)
Aalborg	Sankt Markus	2.77 (1.75, 4.09)
Aalborg	Vor Frelsers	2.79 (1.45, 4.72)
Aalborg	Vesterkær	2.84 (1.65, 4.44)
**Aalborg**	**Skalborg**	**2.85 (1.94, 4.02)**
Aalborg	Hammer	2.89 (1.69, 4.61)
**Aalborg**	**Vor Frue**	**2.89 (1.88, 4.21)**
Aalborg	Lillevorde	2.96 (1.64, 4.98)
Aalborg	Storvorde	2.96 (1.70, 4.75)
Aalborg	Gistrup	3.02 (1.85, 4.62)
Aalborg	Hals	3.10 (1.76, 4.91)
**Aalborg**	**Sønder Tranders**	**3.12 (2.27, 4.21)**
**Aalborg**	**Hans Egedes**	**3.15 (2.03, 4.55)**
**Aalborg**	**Lindholm**	**3.19 (2.09, 4.58)**

**Aalborg**	**Dall**	**3.20 (1.89, 5.13)**
**Aalborg**	**Romdrup-Klarup**	**3.37 (2.21, 4.89)**
**Aalborg**	**Vodskov**	**3.41 (2.19, 4.99)**
**Aalborg**	**Hvorup**	**3.51 (2.38, 4.93)**
**Aalborg**	**Rørdal**	**3.85 (1.94, 6.74)**
**Aalborg**	**Nørre Tranders**	**4.09 (2.81, 5.72)**
**Aalborg**	**Vejgård**	**4.13 (3.04, 5.41)**
Brønderslev	Ørum	1.93 (1.16, 3.00)
Frederikshavn	Bangsbostrand	2.01 (1.23, 2.99)
Helsingør	Sthens	1.98 (1.28, 2.90)
Helsingør	Gurre	2.05 (1.19, 3.29)
Helsingør	Sankt Mariæ	2.17 (1.28, 3.36)
Helsingør	Sankt Olai	2.26 (1.38, 3.39)
Helsingør	Hellebæk	2.39 (1.53, 3.53)
**Helsingør**	**Vestervang**	**3.04 (2.10, 4.19)**
Kalundborg	Røsnæs	3.06 (1.12, 6.45)
Kerteminde	Kølstrup	2.11 (1.17, 3.52)
Kerteminde	Kerteminde-Drigstrup	2.25 (1.36, 3.40)
Kerteminde	Marslev	2.29 (1.40, 3.53)
**Kerteminde**	**Munkebo**	**3.14 (2.00, 4.65)**
Jammerbugt	Aaby	2.10 (1.34, 3.13)
Jammerbugt	Biersted	2.16 (1.24, 3.40)
Odense	Paarup	1.82 (1.25, 2.52)
Odense	Fraugde	1.89 (1.21, 2.79)

Odense	Hans Tausens	1.91 (1.25, 2.78)
Odense	Næsbyhoved-Broby	1.94 (1.24, 2.88)
Odense	Vollsmose	1.97 (1.23, 2.96)
Odense	Vor Frue	2.01 (1.25, 3.03)
Odense	Lumby	2.01 (1.31, 2.94)
Odense	Sankt Hans	2.05 (1.17, 3.32)
Odense	Munkebjerg	2.08 (1.35, 2.99)
Odense	Hjallese	2.09 (1.46, 2.88)
Odense	Dalum	2.12 (1.32, 3.19)
Odense	Næsby	2.12 (1.36, 3.13)
Odense	Stige	2.18 (1.32, 3.42)
Odense	Åsum	2.30 (1.45, 3.54)
Odense	Fredens	2.33 (1.69, 3.12)
Odense	Tornbjerg	2.52 (1.62, 3.70)
Odense	Seden	2.57 (1.57, 3.91)
Odense	Korsløkke	2.52 (1.76, 3.46)
Odense	Agedrup	2.93 (1.71, 4.55)

Table B1. The posterior mean of the smoothed parish-to-national IRR for the 74 parishes flagged with an excess risk of more than 25% with a 95% posterior probability threshold. Parishes that were also flagged considering elevated incidence rates of more than 100% are written in bold. Abbreviations: IRR, incidence rate ratio; CI credible interval

Appendix – references

1. Chan CK, Feinstein AR, Jekel JF, Well CK. The value and hazards of standardization in clinical epidemiologic research. J. Clin. Epidemiol. . 1988;41:1125–34.

2. Leroux B, Lei X, Breslow N. Estimation of Disease Rates in Small Areas: A New Mixed Model for Spatial Dependence. In: Statistical Models in Epidemiology, the Environment and Clinical Trials. Springer Verlag, New York; 2000. p. 179–91.

3. Lee D. CARBayes: An R Package for Spatial Areal Unit Modelling with Conditional Autoregressive Priors. J. Stat. Softw. . 2013;55(13):1–24.

4. Klimadatastyrelsen. Det Danske Kvadratnet. [Internet]. [cited 2025 Mar 12]. Available from: https://dataforsyningen.dk/data/2850
